# Association between food insecurity and chronic noncommunicable diseases in Brazil: a systematic review

**DOI:** 10.1590/1980-549720240041

**Published:** 2024-08-12

**Authors:** Jackson Silva Lima Laurentino, Renatha Celiana da Silva Brito, Rônisson Thomas de Oliveira-Silva, Amanda Soares, Thaís da Conceição Pereira, Elisiandre Martins de Lima, Ana Beatriz Macêdo Venâncio dos Santos, Poliana de Araújo Palmeira

**Affiliations:** I Universidade Federal da Paraíba, Graduate Program in Nutrition Sciences – João Pessoa (PB), Brazil.; II Universidade Federal do Rio Grande do Norte, Graduate Program in Public Health – Natal (RN), Brazil.

**Keywords:** Food insecurity, Chronic noncommunicable diseases, Brazil, Review

## Abstract

**Objective:**

To analyze the association of food insecurity (FI) with chronic noncommunicable diseases (NCDs) in the Brazilian context.

**Methods:**

The review protocol was registered with the International Prospective Register of Systematic Reviews (PROSPERO). The searches were conducted in LILACS and PubMed databases (September/2022). Observational studies carried out in the Brazilian population published since 2003 were included, in which: (1) the association of FI with NCDs was analyzed; and (2) the Brazilian Food Insecurity Scale was used. Studies on pregnant women and those that associated FI with cancer, sexually transmitted infections, and musculoskeletal and respiratory diseases were excluded. The studies were subjected to methodological quality assessment.

**Results:**

A total of 27 cross-sectional studies were included; nine used secondary data from national surveys, and the others used primary data. An association between FI and overweight and obesity in different age groups was verified in the studies.

**Conclusion:**

The included articles did not produce evidence on other NCDs of interest to health in Brazil such as diabetes and high blood pressure. However, they corroborate the already-known relationship between obesity and FI. Studies on the topic, with a longitudinal design, should be encouraged.

## INTRODUCTION

Food Insecurity (FI) is expressed by concern, fear, uncertainty, and deprivation in access to quality food in sufficient quantity to maintain a healthy life^1^. According to the Food and Agriculture Organization of the United Nations (FAO) 2022 report, it is estimated that between 702 and 828 million people experienced hunger in 2021, totaling around 9.8% of the world population^2^. In Brazil, high rates of FI are observed, with data showing that around 125.2 million Brazilians were suffering from some degree of FI and 33 million were facing food deprivation in 2022^3^.

Exposure to FI is associated with negative health outcomes, such as poor nutrition and Chronic Noncommunicable Diseases (NCDs), representing one of the most important public health issues to be faced^4^. In this sense, FI has been associated with important metabolic risk factors and NCDs such as overweight and obesity^5,6^, dyslipidemia^7^, diabetes (DM)^8^, and systemic arterial hypertension (SAH)^9^. The deprivation and difficulty accessing healthy foods among people on FI lead to lower consumption of fruits and vegetables and an increase in the consumption of lower-cost foods with high calorie density, such as processed and ultra-processed foods, generating malnutrition. This factor contributes to the development of health problems such as NCDs^10,11^.

Brazil left the United Nations (UN) Hunger Map in 2014, through Food and Nutritional Security strategies employed since the mid-1990s. However, in 2018, data from the Consumer Expenditure Survey^12^ indicated the increase in FI in Brazil. Conversely, the FAO report, published in 2022, showed the country's return to the hunger map, as a result of the crisis in the political, social, and economic spheres linked to the new coronavirus (COVID-19) pandemic^2^. Added to the scenario of FI evolution in Brazil is the increase in NCDs in the country, which corresponded to around 76% of total deaths in 2019^13^. The increase in NCDs rates in Brazil has been attributed to exposure to risk factors related to lifestyle, such as physical inactivity, alcohol abuse, tobacco use, overweight, and unhealthy eating habits such as low consumption of fruits and vegetables and high consumption of processed and ultra-processed foods. These factors are combined with social inequalities and health inequities^13^, generating progressive and worrying scenarios of an increase in NCDs in Brazil.

The Brazilian Food Insecurity Scale (*Escala Brasileira de Insegurança Alimentar* – EBIA) is the main tool used to measure and monitor FI in Brazil^1,14^, consisting of a scale of experience with food deprivation, validated for the Brazilian population since 2004^14^. Since its validation, EBIA has been incorporated into the main national surveys, such as the National Household Sample Survey (*Pesquisa Nacional por Amostra de Domicílios* – PNAD)^15,16^ and, more recently, the Consumer Expenditure Survey (*Pesquisa de Orçamentos Familiares –* POF, 2017–2018)^12^ and the National Survey on Food Insecurity within the COVID-19 context (*Inquérito de Insegurança Alimentar no Contexto da Pandemia da Covid-19* – VIGISAN)^3^.

However, despite the accumulation of Brazilian studies on FI, its association with NCDs is not yet well documented in the population. Therefore, the objective of this review was to analyze the association of FI with NCDs in the Brazilian context.

## METHODS

This systematic review is registered with the International Prospective Register of Systematic Reviews (PROSPERO), under number CRD42023453178, and followed the recommendations of the Preferred Reporting Items for Systematic Reviews and Meta-Analysis (PRISMA)^17^.

### Search strategy

The bibliographic search was carried out in the two main indexing databases of Brazilian scientific production, the Latin American and Caribbean Health Sciences Literature (LILACS) and the National Library of Medicine – PubMed (date of the last query: September 14, 2022). The following terms were developed by the authors in the databases: *"Food Supply"* (MeSH Terms); OR "*Food Insecurity*" (MeSH Terms); OR "*Food Security*" (MeSH Terms); OR "*Household Food Security*" (Title/Abstract); OR "*Household Food Insecurity*" (Title/Abstract); AND "*Chronic Disease*" (MeSH Terms); OR "*Noncommunicable diseases*" (MeSH Terms); OR "*Overnutrition*" (MeSH Terms); OR "*Obesity*" (MeSH Terms); OR "*Glucose Metabolism Disorders*" (MeSH Terms); OR "*Lipid Metabolism Disorders*" (MeSH Terms); OR "*Hypertension*" (MeSH Terms); OR "*Body Mass Index*" (MeSH Terms); OR "*Health Status*" (MeSH Terms); OR "*Risk Factors*" (MeSH Terms); OR "*Pulmonary Disease, Chronic Obstructive*" (MeSH Terms)*;* OR "*Renal Insufficiency, Chronic*" (MeSH Terms); AND "*Brazil*" (MeSH Terms); OR "*Brazil*" (Title/Abstract); OR "*Brazilian*" (Title/Abstract)*.*

The search included studies published between 2003 (EBIA validation in Brazil) and September 2022 (final data search), in Portuguese, English, and Spanish. After the bibliographic search, duplicate articles were excluded with the aid of the EndNote software.

#### Inclusion/exclusion criteria and risk of bias assessment

The inclusion criteria were applied in two stages. First, two independent researchers evaluated the title and abstract of the articles, according to the following inclusion criteria:

1. Studies that analyzed the association (through association tests) of FI with NCDs or FI with risk indicators for NCDs;
2. Studies carried out in the Brazilian population including all age groups of children, adolescents, adults, and older adults;
3. Studies that used EBIA to measure FI (EBIA has dichotomous questions that adapt to family composition, with eight questions for households without individuals <18 years old and 14 questions for households >18 years old, both were included);
4. Studies that used validated measures for diagnosing NCDs and risk indicators for NCDs; and
5. Observational studies: cross-sectional and longitudinal.

In the second stage, the articles that met the inclusion criteria were read in full and evaluated for compliance with the criteria of the Quality Assessment Tool for Quantitative Studies (QATFQS)^18^, which classifies studies according to their quality as "strong," "moderate," and "weak." This instrument was chosen because it is relevant for the evaluation of cross-sectional observational studies. Studies with a "weak" classification were excluded.

Methodological, qualitative, and quantitative studies with an ecological or case-control design did not meet the inclusion criteria. Studies on pregnant women and those that associated FI with sexually transmitted infections, cancer, musculoskeletal diseases (arthritis, osteoporosis), and respiratory diseases (asthma) were also excluded. In addition, preprint articles were excluded.

In the inclusion stage, agreement between researchers was assessed using the Kappa index^19^. Agreement was determined when both researchers chose to exclude or include the same article.

#### Data extraction

Data extraction was carried out by two independent researchers. Information to be extracted was previously established by the researchers, namely: authors; year of publication; study objective; study location (city/state); study design; study population and year of data collection; type of sample (probabilistic and non-probabilistic); data source (primary or secondary); type of statistical analyses; main results on the association of FI with NCDs; hypotheses and justifications used by the authors to explain the association of FI with NCDs.

The hypotheses and justifications used by the authors to explain the association of FI with NCDs were used to propose a conceptual model on the paths of association between FI and NCDs.

During the data extraction process, it was noted that two of the included articles^20,21^ used EBIA responses as a continuous variable for the analyses, which is not appropriate. These, therefore, were excluded. The results were synthesized and presented in a descriptive way.

## RESULTS

**Figure 1:**
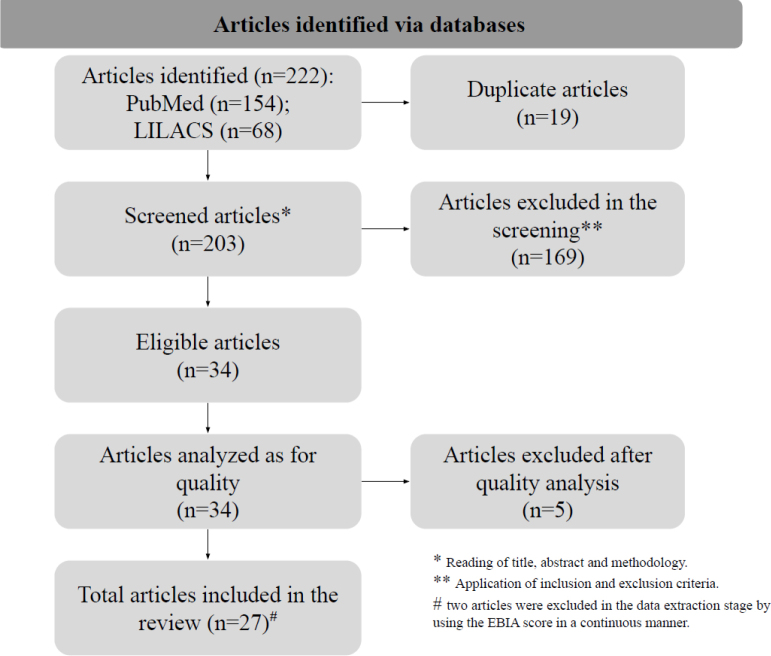
Flowchart of the selection process of studies included in the systematic review on food insecurity and chronic noncommunicable diseases in Brazil, 2023.

In the initial search, we identified 222 articles. With the application of the inclusion and exclusion criteria, 188 articles were excluded (19 due to duplication and 169 for not meeting the inclusion criteria). After quality assessment, this review included 27 articles (Figure 1). In the inclusion stage, the Kappa index was 1.0, indicating perfect agreement.

Among the 27 articles, nine were carried out with national samples of the population. The other studies used regional samples, with n=9 in the Northeast region, n=5 in the Southeast, n=2 in the Midwest, and n=2 in the South (Supplementary Material).

The articles were published between 2012 and 2022 and the data were collected between 2005 and 2019. All analyzed articles have a cross-sectional design, 66.7% (n=18) used primary data and 33.3% (n=9) used secondary data from two national surveys: the National Survey of Demography and Health (*Pesquisa Nacional de Demografia e Saúde* – PNDS) (n= 8) and the Consumer Expenditure Survey – POF (n=1). Most studies used a probabilistic sample (85.2%).

The population groups included in the studies were: adolescents (n=8), children (n=7), women with children (n=4), adults (n=4), adult women (n=2), adults and older adults (n=1), older adults (n=1), and adults and older adults diagnosed with DM and/or SAH users of a Health Center (*Unidade Básica de Saúde* – UBS) (n=1).

Risk indicators for NCDs were: body mass index (BMI) (n=21), weight-for-age index (n=2), weight-for-height index (n=2), waist circumference (WC) (n=2), blood pressure (BP) (n=1), and capillary triglycerides analysis (n=1).

To analyze the associations of FI with risk indicators for NCDs (Table 1)^5,9,22–33^, most authors applied multivariate analysis models: Poisson Regression (n=12), Logistic Regression (n=7), Linear Regression (n=3), and Structural Equation Model (n=1). In five articles, the association was performed using the chi-square test (χ^2^ *test*).

**Table 1:** General characteristics and main results of the studies included in the systematic review that demonstrated associations between food insecurity and chronic noncommunicable diseases, 2023.

Study	Study population and location	Data source and sample	Statistical analysis	Main results	QATFQS
Kac et al.^22^	Female teenagers. Brazil (urban and rural areas).	Secondary data and probabilistic sample (PNDS).	Multivariate Poisson regression. Association between FI (FS x mild FI x moderate FI x severe FI) and overweight in female adolescents. Overweight: BMI-for-age (categorical variable: BMI ≥85th percentile for adolescents aged 15–18 years and BMI≥25 kg/m^2^ for >19 years old) (measurement taken).	Severe FI was associated with overweight in female adolescents. Adjusted PR (95%CI): 1.96 (1.18–3.27) p=0.007.	Strong
Santos et al.^24^	Adolescents. Pernambuco (PE) (urban and rural areas).	Primary data and probabilistic sample.	Poisson regression. Association between mild FI (FS/mild FI x moderate/severe FI) and overweight in adolescents. Overweight: BMI-for-age (categorical variable: > +1 Z-score and ≤ +2 Z-score) (measurement taken).	Mild FI (FS+mild FI) was associated with overweight in adolescents (10 to 19 years of age). Adjusted PR (95%CI): 1.08 (1.0–1.17) p=0.03.	Strong
Santana et al. ^23^	Adolescents. Duque de Caxias (RJ).	Primary data and probabilistic sample.	Simple logistic regression. Association between FI (FS x mild FI x moderate/severe FI) and overweight in male adolescents. Overweight (overweight + obesity): BMI-for-age (categorical variable: overweight: ≥Z-score=+1 and <Z-score=+2; and obesity: ≥Z-score=+2) (measurement taken).	Moderate/severe FI was associated with overweight in male adolescents. p<0.05.	Strong
Schlüssel et al.^5^	Female adolescents and adult women. Brazil (urban and rural areas).	Secondary data and probabilistic sample (PNDS).	Poisson regression. Association between FI (FS x mild FI x moderate FI x severe FI) and overweight in female adolescents. Association between severe FI and obesity in adult women. Overweight: BMI-for-age (categorical variable: BMI ≥85th percentile for adolescents aged 15–18 years and BMI≥25 kg/m^2^ for >19 years old) (measurement taken). Obesity: BMI (categorical variable: BMI ≥ 30 kg/m^2^) (measurement taken).	Overweight was associated with severe FI in female adolescents. Obesity was associated with moderate FI in adult women. Female teenagers: PR (95%CI): 1.96 (1.18–3.27) Adult women: PR (95%CI): 1.49 (1.17–1.90)	Moderate
Pequeno et al.^26^	Adults and older adults. Natal (RN).	Primary data and probabilistic sample.	χ^2^ test. Association between FI (FS x FI) and overweight in adults and older adults. Overweight: BMI (categorical variable: BMI ≥25 kg/m^2^) (measurement taken).	FI was associated with overweight in adults and older adults. p=0.046.	Moderate
Costa et al.^25^	Women. State of Alagoas (urban and rural areas).	Primary data and probabilistic sample.	Poisson regression. Association between FI (FS x FI) and overweight. Overweight: BMI (categorical variable: BMI ≥25 kg/m^2^) (measurement taken).	Overweight was associated with FI in women. Adjusted PR (95%CI): 1.14 (1.07–1.21) p<0.001.	Strong
Velásquez-Melendez et al.^28^	Women. Brazil (urban and rural areas).	Secondary data and probabilistic sample (PNDS).	Multivariate Poisson regression. Association between FI (FS x moderate FI) and obesity in women. Obesity: BMI (categorical variable: BMI ≥30 kg/m^2^) (measurement taken).	Moderate FI was associated with obesity in women. Adjusted PR (95%CI): 1.49 (1.17–1.90) p= 0.010.	Strong
Dias et al.^29^	Adults. Rio Grande (RS) (urban area).	Primary data and probabilistic sample.	Poisson regression, with robust adjustment for variance. Association between FI (yes or no) and households managed by obese individuals. Obesity: BMI (categorical variable: BMI ≥30 kg/m^2^) (self-reported measurement).	Obesity was associated with FI in household heads. Adjusted PR (95%CI): 1.39 (1.13–1.71) p=0.031.	Strong
Souza and Marín-León^30^	Older adults. Campinas (SP), 2019.	Primary data and probabilistic sample.	Multivariate multinomial logistic regression. Association between mild FI (FS x mild FI) and obesity in older adults. Obesity: BMI (categorical variable: BMI ≥28 kg/m^2^) (measurement taken).	Obesity was associated with mild FI in older adults. OR (95%CI): 2.01 (1.04–3.87) p=0.036.	Moderate
Domingos et al.^27^	Adults. Brazil (urban and rural areas).	Secondary data and probabilistic sample (POF-IBGE).	Multivariate multinomial logistic regression. Association between FI (FS x mild FI x moderate FI x severe FI) and obesity in women. Obesity: BMI (categorical variable: BMI ≥30 kg/m^2^) (self-reported measurement).	Obesity was associated with all levels of FI in women. OR: >1.00. p<0.05.	Strong
Gubert et al.^31^	Women with children. Brazil (urban and rural areas).	Secondary data and probabilistic sample (PNDS).	Hierarchical multivariable logistic regression. Association between FI (FS x mild FI x moderate FI x severe FI) and the presence of the Double Burden of Malnutrition. Double burden of malnutrition: BMI for mothers (categorical variable: BMI ≥25 kg/m^2^), short stature for children (categorical variable: height-for-age: ≤2 Z-scores) (measurements taken).	Severe FI was associated with the presence of the Double Burden of Malnutrition. Adjusted OR (95%CI): 3.33 (1.41–7.84)	Strong
Vicenzi et al.^32^	Children >5 years of age. São Leopoldo (RS).	Primary data and non-probabilistic sample.	Poisson regression, with robust adjustment for variance. Association between FI (yes or no) and lower probability of overweight in schoolchildren. Overweight: BMI-for-height (categorical variable: BMI-for-height: >1 Z-score) (measurement taken).	FI was associated with a lower probability of overweight in schoolchildren. Adjusted PR (95%CI): 0.78 (0.64–0.96)	Moderate
Poblacion et al.^33^	Children <5 years of age. Brazil (urban and rural areas).	Secondary data and probabilistic sample (PNDS).	Poisson regression, with robust adjustment for variance. Association between FI (FS x moderate/severe FI) and underweight in children <5 years old. Underweight: weight-for-age (categorical variable: weight-for-age: ≤2 Z-score) (measurement taken).	Underweight was associated with moderate/severe FI in children <5 years. Adjusted PR (95%CI): 1.4 (1.1–1.7) p=0.008.	Strong
Vasconcelos et al.^9^	Adults and older adults diagnosed with SAH and/or DM seen at UBS. Maceió (AL).	Primary data and probabilistic sample.	Simple logistic regression. Association between FI (yes or no) and lower prevalence of central obesity and hypertriglyceridemia in UBS users. Central obesity: WC (categorical variable: yes or no) (measurement taken). Hypertriglyceridemia: Triglycerides >200 mg/dL (categorical variable: yes or no) (measurement taken).	Lower prevalence of central adiposity and hypertriglyceridemia in UBS users was associated with FI. Hypertriglyceridemia: OR (95%CI): 0.74 (0.67–0.82), p=0.001. Central adiposity: OR (95%CI): 0.12 (0.29–0.52), p=0.004.	Strong

All included articles were cross-sectional. QATFQS: Quality Assessment Tool For Quantitative Studies; FI: household food insecurity measured by the Brazilian Food Insecurity Scale; SA: household food security measured by the Brazilian Food Insecurity Scale; BMI: body mass index; PR: Prevalence ratio; CI: confidence interval; p: p-value (statistical significance p<0.05); PNDS: National Survey of Demography and Health; POF: Consumer Expenditure Survey; IBGE: Brazilian Institute of Geography and Statistics; OR: odds ratio; DM: diabetes mellitus; SAH: systemic arterial hypertension; UBS: Health Center; CC: waist circumference.

For analysis purposes, the included articles categorized EBIA into: 2 categories (Food Security [FS] x FI, 9 articles; FS/mild FI x moderate/severe FI, 4 articles; FS x moderate/severe FI, 2 articles; FS x moderate FI, 1 article; FS x mild FI, 1 article; FS/mild/moderate FI x severe FI, 1 article), 3 categories (FS x mild FI x moderate/severe FI, 3 articles), and 4 categories (FS x mild FI x moderate FI x severe FI, 6 articles).

Regarding the association of FI with NCDs, in Table 1 we present a summary of the general characteristics and main results of the included articles that presented associations. According to the studies, there was an association of FI with overweight and overweight/obesity in different age groups of the life cycle and between men and women, assessed mainly through BMI. As for how the FI variable was used in the analyses, 18 studies used it as an exposure variable/risk factor^5,22–24,26–28,31,32,34–42^ and 9 studies as an outcome variable^9,25,29,30,33,43–46^.

In adolescents, authors of the studies found an association between overweight and severe FI^5^ and an association between severe FI^22^, moderate/severe FI^23^, and FS/mild FI^24^ and overweight. An association between overweight and FI (FS x FI) was also found in women^25^ and in adults and older adults, regardless of sex^26^.

Regarding obesity, an association of this condition was found with all levels of FI^27^ and moderate FI^5^ in women. Furthermore, an association between the moderate FI variable^28^ and obesity in women was observed. An association between obesity and FI was also identified among those responsible for the household^29^ and it was associated with mild FI in older adults^30^. In addition, we noticed an association between severe FI and the double burden of malnutrition, that is, an overweight mother and stunted children in the same household^31^.

Conversely, the lower prevalence of abdominal obesity and hypertriglyceridemia in users with hypertension and/or DM seen at UBS was associated with FI^9^, and the lower prevalence of overweight and obesity was associated with moderate and severe FI in men^29^. FI was associated with lower probability of being overweight in schoolchildren over 5 years of age^32^, and underweight was associated with moderate/severe FI in children under 5 years of age^33^.

Among the included articles, 13 did not demonstrate an association between FI and indicators related to NCDs^34–46^, eight of which were carried out with children and adolescents^37–43,46^. These articles are presented in Supplementary Material.

**Figure 2:**
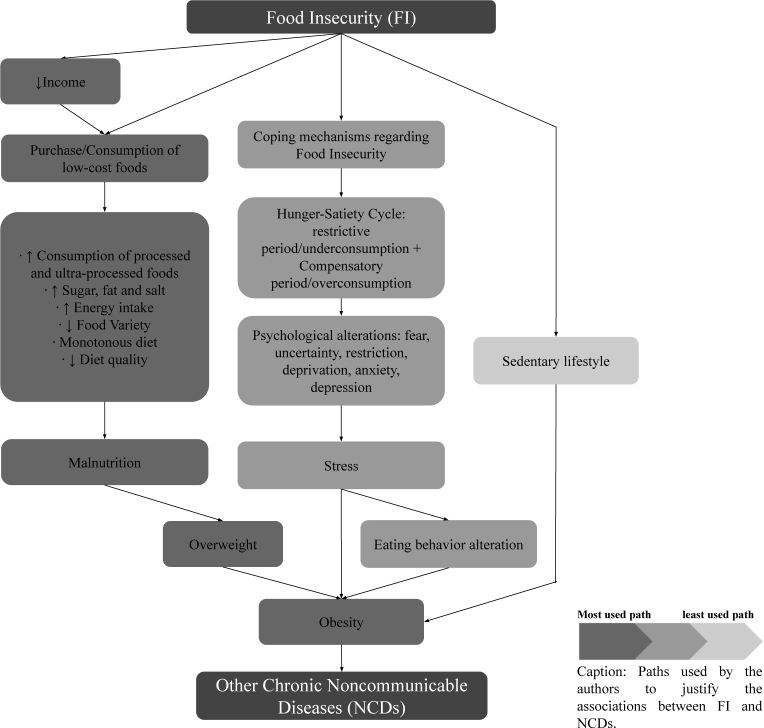
Conceptual model of the paths used in the included articles to explain the association of food insecurity with obesity and other chronic noncommunicable diseases. Systematic review, 2023.

With the hypotheses and justifications used by the authors of the included studies to explain the association of FI with obesity, we proposed a theoretical model with the main paths of this association, presented in Figure 2.

## DISCUSSION

The main objective of this systematic review was to use the successful history of studies on FI to determine and document evidence on the association of FI with NCDs in the context of the Brazilian population. According to the findings, there is an association between FI and overweight in adolescents, adults, and older adults of both sexes and with obesity in women and older adults.

The association of FI with NCDs has been addressed in systematic reviews. Miguel et al.^47^ studied the relationship between FI and cardiometabolic risk factors in adults and older adults. The review included 11 studies with proxy measures for NCDs, showing the existence of a direct association between FI and conditions such as overweight, hypertension, DM, dyslipidemia, and stress. Regarding DM, Abdurahman et al.^48^ identified that FI was associated with type 2 DM; however, they highlighted that in Canada the choice of the FI measurement instrument influences the effect on the chance of developing DM. Conversely, Beltrán et al.^49^ found no association between DM and FI. For cardiovascular diseases, Liu and Eicher-Miller^50^ observed an association of FI with hypertension, coronary heart disease, and heart attack in North Americans over 17 years of age.

Beltrán et al.^51^ found an association between FI in adults with self-reported hypertension, but this association was not found when the authors made the diagnosis by measuring blood pressure or reviewing medical records. Arenas et al.^52^ studied the association of FI with dyslipidemia, and the results did not show an association between FI and dyslipidemia measured by laboratory lipid analysis, but individuals on FI were more likely to self-report previous diagnoses of dyslipidemia. Finally, on the international scene, the association of FI with obesity has also been documented in previous systematic reviews^53–56^.

In this review, dedicated to studying the Brazilian population, despite the broad search spectrum, the significant majority of available studies addressed nutritional status and analyses of the condition of overweight and obesity related to FI, thus highlighting the small volume of studies with a population at risk or affected by hypertension, DM, dyslipidemia, and cardiovascular diseases. In addition, we observed little diversity of risk indicators, with most studies using only BMI.

As noted in this review, authors of other reviews have documented the association of FI with overweight/obesity in the Brazilian population. Morais et al.^57,58^ observed an association between FI and overweight and obesity in the Brazilian population; however, with regard to the rural population, Trivellato et al.^59^ found no association between nutritional status (overweight, underweight, and/or chronic malnutrition) and FI.

In Figure 2 we illustrate a theoretical model containing the main paths followed by the authors of the included studies to explain the association of FI with obesity, namely:

1. Malnutrition;
2. Coping mechanisms and coexistence with FI; and
3. Sedentary lifestyle.

The path most used by the authors to explain this association was malnutrition, that is, in families that experience FI there is the purchase and consumption of low-cost foods, such as processed and ultra-processed foods, and, consequently, an increase in energy, sugar, fat, and salt intake.

The second path most used by the authors is based on the existence of coping mechanisms and coexistence with FI within families, such as the hunger-satiety cycle, characterized by restrictive periods of underconsumption followed by compensatory periods of overconsumption. This mechanism predisposes metabolic and psychological alterations, such as fear, uncertainty, restriction, deprivation, anxiety, and depression, configuring acute and chronic stress conditions that alter eating behavior and favor the development of obesity.

The least used path was sedentary lifestyle, which theorizes that the state of physical inactivity of people on FI, resulting from their socioeconomic status, favors the development of obesity. In the three paths used in the included articles, obesity appears as a factor that mediates the development process of other NCDs.

Accordingly, at an international level, researchers also use these paths to explain the association^53,54,60,61^. The relationship between FI and malnutrition is documented in the literature. Authors describe that the presence of FI is accompanied by less variety of foods, greater energy intake, and monotonous diets, that is, it is not just the absence of food, but also access to low-quality foods^47,57,62,63^.

Researchers point to the presence of obesity as the main path and mediator that connects FI to the development of other NCDs such as DM and SAH. Nonetheless, authors highlight the importance of analyzing the potential negative effects of FI on health, regardless of obesity^50,64,65^.

It is worth highlighting the need to deepen the debate on the association of FI with NCDs, incorporating into studies variables related to social inequalities, and with regard to access to health, working conditions and the food environment, topics that contribute to expanding the context of discussion and the network of relevant factors for the debate, both on FI and NCDs — especially in the Brazilian scenario, marked by profound social and regional inequality.

Therefore, the association between FI and NCDs is complex. The emergence of a chronic condition, such as obesity, DM, and SAH, results from a broad set of determining factors and is also related to previous and continuous exposure to these determinants. Nevertheless, it is noteworthy that in the studies included in the review, the population's exposure time to FI was not considered, due to the cross-sectional nature of the studies.

Hence, the lack of longitudinal studies that analyze the development of NCDs from exposure to FI is an important scientific gap. Conversely, according to the studies, an association between FI and the risk/presence of NCDs was established, that is, individuals who live with these conditions also suffer from food deprivation, a context that is expressed as an important limiting factor in the implementation and adoption of safety measures of care and treatment of these chronic conditions.

It should be noted that, at a global level, FI measurement methods differ between countries/regions, especially in the FI reference time, also making it difficult to compare evidence. Another complexity related to the analysis of FI and NCDs is the social and economic context of each country, in such a way that differences in agrifood systems, development levels, and the state of nutritional transition must be considered in future studies and for comparison with observed results in Brazil.

The articles included in this review did not produce evidence on other NCDs of interest to public health in Brazil, such as DM and SAH, highlighting an important scientific gap and the need to develop a research agenda on the topic that prioritizes longitudinal studies, aiming to produce evidence of causality in the relationship between FI and NCDs.

The evidence produced here reinforces that access to food must be guaranteed, with health-promoting practices, and provides support for focusing public health interventions and programs on combating FI and promoting, preventing, and controlling NCDs. Health services must be aware of the high prevalence of FI in the Brazilian population and how this scenario can impact the care offered to individuals affected by NCDs. Investments in interventions and public programs related to the topic of the association of FI with NCDs are in line with the UN Sustainable Development Goals, assumed by Brazil for the 2030 Agenda.

Therefore, despite its successful history of studies on FI, in Brazil the association of FI with NCDs remains little addressed. The reviewed studies corroborate evidence of an association between FI and overweight in all age groups, and obesity among women and older adults. Therefore, public interventions and a strong agenda to combat hunger combined with health-promoting practices and nutritional care are necessary, especially in Primary Health Care. We suggest investments are made in studies that address the topic of the association of FI with NCDs, in addition to obesity, mainly with a longitudinal design.

## Supplementary Material


