# Supplementary material for: Association between food insecurity and chronic noncommunicable diseases in Brazil: a systematic review
Source: Rev Bras Epidemiol. 2024 Aug 12;27:e240041. doi: 10.1590/1980-549720240041 (PMC11323874; doi:10.1590/1980-549720240041)
Supplement: Supplementary file 1 [file 1980-5497-rbepid-27-e240041-Suppl01.pdf]

Tabela Suplementar - Características gerais e principais resultados dos estudos incluídos na revisão sistemática que não apresentaram associação da IA com as DCNTs. 2023.

Autores (ano)	População e local do estudo	Fonte dos dados e amostra	Análise estatística	Resultados principais	QATFQS
Gubert *et al.*, (2016)[22]	Crianças < 5 anos. Brasil, (áreas urbanas e rurais).	Dados secundários e amostra probabilística (PNDS).	Regressão logística multivariável hierárquica. Associação entre IA (SA/IA leve/moderada x IA grave) e sobrepeso em crianças < 5 anos. Sobrepeso: peso-para-estatura (variável categórica: >+ 2 escore-Z) (medida aferida).	Não foi encontrada associação estatística.	Moderada
Domingos *et al.*, (2016)[35]	Adultos. Duque de Caxias/RJ (área urbana).	Dados primários e amostra probabilística.	Regressão multivariada de Poisson. Associação entre hipertensão arterial e IA (SA x IA moderada/grave). Hipertensão arterial: Pressão arterial sistólica ≥140 mmHg e/ou pressão arterial diastólica ≥90 mmHg (variável categórica: sim ou não) (medida aferida).	Não foi encontrada associação estatística.	Forte
Freitas *et al.*, (2018)[36]	Adultos. Alegre/ES (áreas urbanas e rurais).	Dados primários e amostra probabilística.	Modelo de regressão linear. Associação entre IA (sim ou não) e Adiposidade Central. Adiposidade central: CC risco (variável categórica: sim ou não) (medida aferida).	Não foi encontrada associação estatística.	Forte

Géa-Horta *et al.*, (2017)[37]	Mulheres com filhos. Brasil, (áreas urbanas e rurais).	Dados secundários e amostra probabilística (PNDS).	Modelagem de equações estruturais. Associação entre IA (SA x IA) e IMC em mães e crianças. IMC em mães: IMC (variável contínua). IMC em crianças: IMC-para-idade escore-Z (variável contínua). (medidas aferidas).	Não foi encontrada associação estatística.	Forte
Santos *et al.*, (2020)[38]	Mulheres com filhos. Bayeux/PB e Cabedelo/PB.	Dados primários e amostra probabilística.	Teste de qui-quadrado. Associação entre IA (SA/IA leve x IA moderada/grave) e excesso de peso em mulheres mães. Excesso de peso: IMC (variável categórica: IMC ≥ 25) (medida aferida).	Não foi encontrada associação estatística.	Forte
Soares *et al.*, (2020)[39]	Mulheres beneficiadas pelo programa "Minha casa, Minha vida" Petrolina, PE.	Dados primários e amostra não-probabilística.	Regressão logística simples. Associação entre IA (SA x IA leve x IA moderada x IA grave) e excesso de peso. Excesso de peso: IMC (variável categórica: sobrepeso e obesidade) (medida aferida).	Não foi encontrada associação estatística.	Moderada
Guerra *et al.*, (2013)[40]	Adolescentes. Mato Grosso/MS.	Dados primários e amostra probabilística.	Regressão de Poisson Múltipla. Associação entre IA (SA/IA leve x IA moderada/grave) e Estado nutricional. Estado nutricional: IMC (variável categórica).	Não foi encontrada associação estatística.	Forte

			(medida aferida).		
Kac *et al*., (2012)[41]	Crianças < 5 anos. Brasil, (áreas urbanas e rurais).	Dados secundários e amostra probabilística (PNDS).	Modelo de regressão linear. Associação entre IA (SA x IA leve x IA moderada/grave) e Peso para estatura e IMC. Peso para altura: variável contínua escore-Z. IMC: IMC-para-idade (variável contínua: escore-Z). (medidas aferidas).	Não foi encontrada associação estatística.	Forte
Lopes *et al*., (2013)[42]	Adolescentes. Duque de Caxias/RJ.	Dados primários e amostra probabilística.	Teste de qui-quadrado Associação entre IA (SA x IA leve x IA moderada/grave) e sobrepeso. Sobrepeso: IMC-para-idade (variável categórica: > +1 escore-Z) (medida aferida).	Não foi encontrada associação estatística.	Forte
Lucena *et al*., (2019)[43]	Crianças de 2 a 8 anos. Maceió/AL.	Dados primários e amostra não probabilística.	Regressão multivariada de poisson. Associação entre IA (SA x IA leve x IA moderada x IA grave) e sobrepeso e obesidade. Sobrepeso: IMC-para-idade (variável categórica: entre + 1 e + 2 escore-Z). Obesidade: IMC-para-idade (variável categórica: ≥ +2 escore-Z). (medida aferida).	Não foi encontrada associação estatística.	Moderada
Pedraza *et al*.,	Crianças < 5 anos.	Dados primários e amostra	Modelo de regressão linear. Associação entre IA (sim ou não) e	Não foi encontrada associação estatística.	Moderada

(2014)[44]	Paraíba/PB.	probabilística.	peso.<br>Peso: peso-para-idade (variável contínua: médias de escore-Z) (medida aferida).		
Cordeiro; Monego; Martins (2014)[45]	Crianças > 5 anos e adolescentes de comunidades quilombolas.<br>Goiás/GO.	Dados primários e amostra probabilística.	Teste de qui-quadrado.<br>Associação entre IA (SA x IA) e sobrepeso.<br>Sobrepeso: IMC-para-idade (variável categórica: escore-Z) (medida aferida).	Não foi encontrada associação estatística.	Forte
Pinheiro *et al.*, (2016)[46]	Adolescentes do sexo feminino e Mulheres adultas em idade reprodutiva.<br>Vitória de Santo Antão/PE (área urbana).	Dados primários e amostra probabilística.	Regressão de poisson bivariada.<br>Associação entre IA (SA/IA leve x. IA moderada/grave) e excesso de peso.<br>Excesso de peso: IMC (variável categórica: sobrepeso + obesidade) (medida aferida).	Não foi encontrada associação estatística.	Forte

Todos os artigos incluídos foram do tipo transversal. QATFQS**:** Quality Assessment Tool For Quantitative Studies. IA: Insegurança alimentar domiciliar mensurada pela Escala Brasileira de Insegurança Alimentar. SA: Segurança alimentar domiciliar mensurada pela Escala Brasileira de Insegurança Alimentar. PNDS: Pesquisa Nacional de Demografia e Saúde. $\chi 2$ test: Teste qui-quadrado. CC: Circunferência da Cintura. IMC: Índice de massa corporal. Mulheres em idade reprodutiva: 15 a 49 anos.

Figura Suplementar. Distribuição dos artigos incluídos na revisão sistemática segundo sua localidade de realização por macrorregião brasileira. 2023.

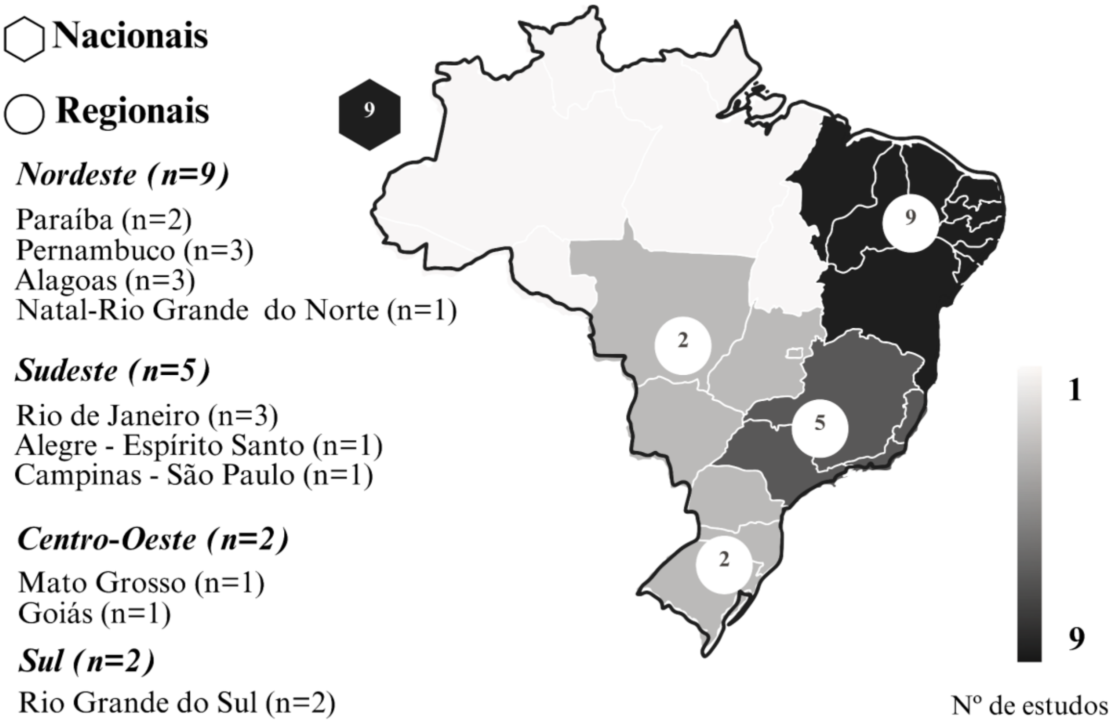

Fonte: Autores (2023).
