# Supplementary material for: Association between food insecurity and chronic noncommunicable diseases in Brazil: a systematic review
Source: Rev Bras Epidemiol. 2024 Aug 12;27:e240041. doi: 10.1590/1980-549720240041 (PMC11323874; doi:10.1590/1980-549720240041)
Supplement: Translation

# Translation: Associação entre insegurança alimentar e doenças crônicas não transmissíveis no Brasil: uma revisão sistemática

**DOI:** 10.1590/1980-549720240041.2

**Authors:** Jackson Silva Lima Laurentino, Renatha Celiana da Silva Brito, Rônisson Thomas de Oliveira-Silva, Amanda Soares, Thaís da Conceição Pereira, Elisiandre Martins de Lima, Ana Beatriz Macêdo Venâncio dos Santos, Poliana de Araújo Palmeira

**Affiliations:** I Universidade Federal da Paraíba, Programa de Pós-Graduação em Ciências da Nutrição – João Pessoa (PB), Brasil.; II Universidade Federal do Rio Grande do Norte, Programa de Pós-Graduação em Saúde Coletiva – Natal (RN), Brasil.

**Keywords:** Insegurança alimentar, Doenças crônicas não transmissíveis, Brasil, Revisão

## Abstract

**Objetivo:**

Analisar a associação da insegurança alimentar (IA) com as doenças crônicas não transmissíveis (DCNTs) no contexto brasileiro.

**Métodos:**

O protocolo da revisão foi registrado no *International Prospective Register of Systematic Reviews* (PROSPERO). As buscas foram realizadas na LILACS e na PubMed (setembro/2022). Foram incluídos estudos observacionais realizados com a população brasileira, publicados a partir de 2003, que: (1) analisam a associação da IA com DCNTs; e (2) utilizam a Escala Brasileira de Insegurança Alimentar (EBIA). Foram excluídos estudos com gestantes e que associam a IA com câncer, infecções sexualmente transmissíveis e doenças musculoesqueléticas e respiratórias. Os estudos foram submetidos à avaliação da qualidade metodológica.

**Resultados:**

Foram incluídos 27 estudos transversais, dentre os quais 9 utilizaram dados secundários de inquéritos nacionais e os demais utilizaram dados primários. Os estudos revelaram associação da IA com sobrepeso e obesidade em diferentes faixas etárias.

**Conclusão:**

Os artigos incluídos não produziram evidências sobre outras DCNTs de interesse para a saúde no Brasil, como a diabetes e a hipertensão arterial. No entanto, contribuem para corroborar a relação já conhecida entre obesidade e IA. Estudos sobre a temática, com desenho longitudinal, devem ser incentivados.

## INTRODUÇÃO

A Insegurança Alimentar (IA) se expressa por preocupação, medo, incerteza e privação no acesso a alimentos de qualidade e em quantidade suficientes para se manter uma vida saudável^1^. De acordo com o relatório da FAO 2022, estima-se que entre 702 e 828 milhões de pessoas vivenciaram a fome em 2021, totalizando cerca de 9,8% da população mundial^2^. No Brasil observam-se elevadas taxas de IA, com dados apontando que cerca de 125,2 milhões de brasileiros encontravam-se em algum grau de IA e 33 milhões enfrentavam privação alimentar em 2022^3^.

A exposição à IA associa-se a desfechos negativos em saúde, como a má nutrição e Doenças Crônicas Não Transmissíveis (DCNTs), representando um dos mais importantes problemas de saúde pública a serem enfrentados^4^. Neste sentido, a IA tem sido associada a fatores de risco metabólicos e DCNTs importantes, como excesso de peso e obesidade^5,6^, dislipidemia^7^, diabetes (DM)^8^ e hipertensão arterial sistêmica (HAS)^9^. A privação e a dificuldade de acesso a alimentos saudáveis entre pessoas em IA levam ao menor consumo de frutas e vegetais e ao aumento do consumo de alimentos de menor custo e alta densidade calórica, como os alimentos processados e ultraprocessados, gerando quadros de má nutrição. Esse fator contribui para o desenvolvimento de agravos à saúde, como as DCNTs^10,11^.

O Brasil saiu do Mapa da Fome da Organização das Nações Unidas (ONU) em 2014, através de estratégias de Segurança Alimentar e Nutricional aplicadas desde meados da década de 1990. Todavia, em 2018, dados da Pesquisa de Orçamento Familiar^12^ sinalizaram o aumento da IA no Brasil. Já o relatório da FAO, publicado em 2022, mostrou o retorno do país ao mapa da fome, em consequência da crise nos campos político, social e econômico, atrelados à pandemia da COVID-19^2^. Soma-se ao cenário de evolução da IA no Brasil o aumento das DCNTs no país, que correspondeu a cerca de 76% do total de óbitos em 2019^13^. O aumento das taxas de DCNTs no Brasil tem sido atribuído à exposição a fatores de risco relacionados a estilo de vida, como inatividade física, consumo abusivo de álcool, uso de tabaco, sobrepeso e hábitos alimentares não saudáveis, como o baixo consumo de frutas e vegetais e o alto consumo de alimentos processados e ultraprocessados. Esses fatores estão aliados às desigualdades sociais e às iniquidades em saúde^13^, gerando cenários progressivos e preocupantes de aumento de DCNTs no Brasil.

A Escala Brasileira de Insegurança Alimentar (EBIA) é a principal ferramenta utilizada para mensurar e monitorar a IA no Brasil^1,14^, sendo uma escala de experiência com a privação alimentar, validada para a população brasileira desde 2004^14^. Desde a sua validação, a EBIA foi incorporada aos principais inquéritos nacionais, a exemplo da Pesquisa Nacional por Amostra de Domicílios (PNAD)^15,16^ e, mais recentemente, a Pesquisa de Orçamentos Familiares – POF (2017–2018)^12^ e o VIGISAN^3^.

Contudo, apesar do acúmulo de estudos brasileiros sobre IA, a sua associação com DCNTs ainda não está bem documentada na população. Dessa maneira, o objetivo desta revisão foi analisar a associação da IA com as DCNTs no contexto brasileiro.

## MÉTODOS

Esta revisão sistemática está registrada no *International Prospective Register of Systematic Reviews* (PROSPERO), sob o número CRD42023453178, e seguiu as recomendações de *Preferred Reporting Items for Systematic Reviews and Meta-Analysis* (PRISMA)^17^.

### Estratégia de busca

A busca bibliográfica foi realizada nas duas principais bases de dados de indexação da produção científica brasileira, a Literatura Latino-Americana e do Caribe em Ciências da Saúde (LILACS) e a Biblioteca Nacional de Medicina – PubMed (data da última consulta: 14 de setembro de 2022). Os seguintes termos foram desenvolvidos pelos autores nas bases de dados: *"Food Supply"* (MeSH Terms); OR "*Food Insecurity*" (MeSH Terms); OR "*Food Security*" (MeSH Terms); OR "*Household Food Security*" (Title/Abstract); OR "*Household Food Insecurity*" (Title/Abstract); AND "*Chronic Disease*" (MeSH Terms); OR "*Noncommunicable diseases*" (MeSH Terms); OR "*Overnutrition*" (MeSH Terms); OR "*Obesity*" (MeSH Terms); OR "*Glucose Metabolism Disorders*" (MeSH Terms); OR "*Lipid Metabolism Disorders*" (MeSH Terms); OR "*Hypertension*" (MeSH Terms); OR "*Body Mass Index*" (MeSH Terms); OR "*Health Status*" (MeSH Terms); OR "*Risk Factors*" (MeSH Terms); OR "*Pulmonary Disease, Chronic Obstructive*" (MeSH Terms)*;* OR "*Renal Insufficiency, Chronic*" (MeSH Terms); AND "*Brazil*" (MeSH Terms); OR "*Brazil*" (Title/Abstract); OR "*Brazilian*" (Title/Abstract)*.*

A busca contemplou estudos publicados entre 2003 (validação da EBIA no Brasil) e setembro de 2022 (busca final de dados), em português, inglês e espanhol. Após a busca bibliográfica, os artigos duplicados foram excluídos com o auxílio do *software* *EndNote.*

#### Critérios de inclusão/exclusão e avaliação do risco de viés

Os critérios de inclusão foram aplicados em duas etapas. Primeiro, dois pesquisadores independentes avaliaram o título e o resumo dos artigos, de acordo com os seguintes critérios de inclusão:

1. Estudos que analisaram a associação (por testes de associação) da IA com as DCNTs ou da IA com indicadores de risco para DCNTs;
2. Estudos realizados na população brasileira incluindo todos os grupos etários de crianças, adolescentes, adultos e idosos;
3. Estudos que utilizam a EBIA para mensurar IA (a EBIA possui questões dicotômicas que se adequam à composição familiar, sendo 8 questões para domicílios sem indivíduos <18 anos e 14 para domicílios com >18 anos, as duas foram incluídas);
4. Estudos que usaram medidas validadas para diagnóstico de DCNTs e indicadores de risco para DCNTs; e
5. Estudos observacionais: transversais, longitudinais.

Na segunda etapa, os artigos que atenderam os critérios de inclusão foram lidos na íntegra e avaliados quanto à conformidade com os critérios do *Quality Assessment Tool For Quantitative Studies* (QATFQS)^18^, que classifica os estudos quanto à sua qualidade, em "forte", "moderada" e "fraca". Esse instrumento foi escolhido por apresentar-se pertinente para a avaliação de estudos observacionais transversais. Os estudos com classificação "fraca" foram excluídos.

Não atenderam aos critérios de inclusão estudos metodológicos, qualitativos e quantitativos com desenho ecológico ou caso controle. Também foram excluídos estudos com gestantes e que associaram a IA com infecções sexualmente transmissíveis, câncer, doenças musculoesqueléticas (artrite, osteoporose) e doenças respiratórias (asma). Os artigos publicados em *preprint* também foram excluídos.

Na etapa de inclusão, a concordância entre os pesquisadores foi avaliada pelo índice de *Kappa* ^19^. A concordância foi determinada quando os dois pesquisadores optaram por excluir ou incluir o mesmo artigo.

#### Extração de dados

A extração dos dados foi realizada por dois pesquisadores independentes. As informações a serem extraídas foram previamente estabelecidas pelos pesquisadores, sendo elas: autores; ano de publicação; objetivo do estudo; local do estudo (cidade/estado); tipo de estudo; população do estudo e ano da coleta de dados; tipo da amostra (probabilística e não probabilística); fonte dos dados (primários ou secundários); tipo de análises estatísticas; principais resultados sobre a associação da IA com DCNTs; hipóteses e justificativas utilizadas pelos autores para explicar a associação da IA com DCNTs.

As hipóteses e justificativas utilizadas pelos autores para explicar a associação da IA com as DCNTs foram utilizadas para a proposição de um modelo conceitual sobre os caminhos da associação da IA com as DCNTs.

Durante o processo de extração dos dados, notou-se que dois dos artigos incluídos^20,21^ utilizaram as respostas da EBIA como variável contínua para as análises, o que não é adequado. Estes, portanto, foram excluídos. Os resultados foram sintetizados e apresentados de maneira descritiva.

## RESULTADOS

A busca inicial identificou 222 artigos. Com a aplicação dos critérios de inclusão e exclusão, foram retirados 188 (19 por duplicidade e 169 por não atenderem os critérios de inclusão). Após a avaliação da qualidade, esta revisão incluiu 27 artigos (Figura 1). Na etapa de inclusão, o índice *Kappa* foi de 1,0, indicando concordância perfeita.

**Figura 1:** Fluxograma do processo de seleção dos estudos incluídos na revisão sistemática sobre insegurança alimentar e doenças crônicas não transmissíveis no Brasil, 2023.

Dentre os 27 artigos, 9 foram realizados com amostras nacionais da população. Os demais estudos usaram amostras regionais, sendo n=9 na região Nordeste, n=5 na Sudeste, n=2 na Centro-Oeste e n=2 na Sul (material suplementar).

Os artigos foram publicados entre 2012 e 2022 e os dados foram coletados entre 2005 e 2019. Todos os artigos analisados apresentam desenho transversal, 66,7% (n=18) utilizaram dados primários e 33,3% (n=9) dados secundários oriundos de dois inquéritos nacionais: a Pesquisa Nacional de Demografia e Saúde – PNDS (n=8) e a Pesquisa de Orçamentos Familiares – POF (n=1). A maioria dos estudos usou amostra probabilística (85,2%).

Os grupos populacionais incluídos nos estudos foram: adolescentes (n=8), crianças (n=7), mulheres com filhos (n=4), adultos (n=4), mulheres adultas (n=2), adultos e idosos (n=1), idosos (n=1) e adultos e idosos com diagnóstico de DM e/ou HAS usuários de Unidade Básica de Saúde – UBS (n=1).

Os indicadores de risco para DCNTs foram: índice de massa corporal (IMC) (n=21), índice de peso-por-idade (n=2), índice de peso-por-altura (n=2), circunferência da cintura (CC) (n=2), pressão arterial (PA) (n=1) e análise capilar de triglicerídeos (n=1).

Para analisar as associações da IA com os indicadores de risco para as DCNTs (Tabela 1)^5,9,22–33^, a maioria dos autores aplicou modelos de análise multivariada: Regressão de Poisson (n=12), Regressão logística (n=7), Regressão linear (n=3), e Modelo de equação estrutural (n=1). Em cinco artigos foi realizada a associação por teste de qui-quadrado (χ^2^ *test*).

**Tabela 1:** Características gerais e principais resultados dos estudos incluídos na revisão sistemática que demonstraram associações da insegurança alimentar com as doenças crônicas não transmissíveis, 2023.

Estudo	População e local do estudo	Fonte dos dados e amostra	Análise estatística	Resultados principais	QATFQS
Kac et al.^22^	Adolescentes do sexo feminino. Brasil (áreas urbanas e rurais).	Dados secundários e amostra probabilística (PNDS).	Regressão multivariada de Poisson. Associação entre IA (SA x IA leve x IA moderada x IA grave) e excesso de peso em adolescente do sexo feminino. Excesso de peso: IMC-para-idade (variável categórica: IMC ≥85 percentil para adolescentes de 15–18 anos e IMC ≥25 kg/m^2^ para >19 anos) (medida aferida).	IA grave foi associada a excesso de peso em adolescente do sexo feminino. PR (IC95%) ajustada: 1,96 (1,18–3,27). p=0,007.	Forte
Santos et al.^24^	Adolescentes. Pernambuco (PE) (áreas urbanas e rurais).	Dados primários e amostra probabilística.	Regressão de Poisson. Associação entre IA leve (SA/IA leve x IA moderada/grave) e excesso de peso em adolescentes. Sobrepeso: IMC-para-idade (variável categórica: > +1 escore-Z and ≤ +2 escore-Z) (medida aferida).	IA leve (SA+IA leve) foi associada a excesso de peso em adolescentes (10 a 19 anos). PR (IC95%) ajustada: 1,08 (1,0–1,17). p=0,03.	Forte
Santana et al. ^23^	Adolescentes. Duque de Caxias (RJ).	Dados primários e amostra probabilística.	Regressão logística simples. Associação entre IA (SA x IA leve x IA moderada/grave) e excesso de peso em adolescentes do sexo masculino. Excesso de peso (sobrepeso + obesidade): IMC-para-idade (variável categórica: sobrepeso: ≥escore-Z=+1 e <escore-Z=+2; e obesidade: ≥escore-Z=+2) (medida aferida).	IA moderada/grave foi associada a excesso de peso em adolescentes do sexo masculino. p<0,05.	Forte
Schlüssel et al.^5^	Adolescentes do sexo feminino e Mulheres adultas. Brasil (áreas urbanas e rurais).	Dados secundários e amostra probabilística (PNDS).	Regressão de Poisson. Associação entre IA (SA x IA leve x IA moderada x IA grave) e excesso de peso em adolescente do sexo feminino. Associação entre IA grave e obesidade em mulheres adultas. Excesso de peso: IMC-para-idade (variável categórica: IMC ≥85 percentil para adolescente de 15–18 anos e IMC ≥25 kg/m^2^ para 19 anos) (medida aferida). Obesidade: IMC (variável categórica: IMC ≥ 30 kg/m^2^) (medida aferida).	Excesso de peso foi associado como IA grave em adolescente do sexo feminino. Obesidade foi associada a IA moderada em mulheres adultas. Adolescentes do sexo feminino: PR (IC95%): 1,96 (1,18–3,27). Mulheres adultas: PR (IC95%): 1,49 (1,17–1,90).	Moderada
Pequeno et al.^26^	Adultos e idosos. Natal (RN).	Dados primários e amostra probabilística.	Teste de χ^2^. Associação entre IA (SA x IA) e sobrepeso em adultos e idosos. Sobrepeso: IMC (variável categórica: IMC ≥25 kg/m^2^) (medida aferida).	IA foi associada a sobrepeso em adultos e idosos. p=0,046.	Moderada
Costa et al.^25^	Mulheres. Estado de Alagoas (áreas urbanas e rurais).	Dados primários e amostra probabilística.	Regressão de Poisson. Associação entre IA (SA x IA) e sobrepeso. Sobrepeso: IMC (variável categórica: IMC ≥25 kg/m^2^) (medida aferida).	Sobrepeso foi associado a IA em mulheres. PR (IC95%) ajustada: 1,14 (1,07–1,21) p<0,001.	Forte
Velásquez-Melendez et al.^28^	Mulheres. Brasil (áreas urbanas e rurais).	Dados secundários e amostra probabilística (PNDS)	Regressão multivariada de Poisson. Associação entre IA (SA x IA moderada) e obesidade em mulheres. Obesidade: IMC (variável categórica: IMC ≥30 kg/m^2^) (medida aferida).	IA moderada foi associada a obesidade em mulheres. PR (IC95%) ajustada: 1,49 (1,17–1,90) p= 0,010.	Forte
Dias et al.^29^	Adultos. Rio Grande (RS) (área urbana).	Dados primários e amostra probabilística.	Regressão de Poisson, com ajuste robusto para variância. Associação entre IA (sim ou não) e domicílios chefiados por indivíduos com obesidade. Obesidade: IMC (variável categórica: IMC ≥30 kg/m^2^) (medida autorreferida).	Obesidade foi associada a IA em chefes de família. PR (IC95%) ajustada: 1,39 (1,13–1,71). p=0,031.	Forte
Souza e Marín-León^30^	Idosos. Campinas (SP).	Dados primários e amostra probabilística.	Regressão logística multinomial multivariada. Associação entre IA leve (SA x IA leve) e obesidade em idosos. Obesidade: IMC (variável categórica: IMC ≥28 kg/m^2^) (medida aferida).	Obesidade foi associada a IA leve em idosos. OR (IC95%): 2,01 (1,04–3,87). p=0,036.	Moderada
Domingos et al.^27^	Adultos. Brasil, (áreas urbanas e rurais).	Dados secundários e amostra probabilística (POF-IBGE).	Regressão logística multinomial multivariada. Associação entre IA (SA x IA leve x IA moderada x IA grave) e obesidade em mulheres. Obesidade: IMC (variável categórica: IMC ≥30 kg/m^2^) (medida autorreferida).	Obesidade foi associada a todos os níveis de IA em mulheres. OR: >1,00. p<0,05.	Forte
Gubert et al.^31^	Mulheres com filhos. Brasil, (áreas urbanas e rurais).	Dados secundários e amostra probabilística (PNDS).	Regressão logística multivariável hierárquica. Associação entre IA (SA x IA leve x IA moderada x IA grave) e a presença de Dupla Carga de Desnutrição. Dupla carga de desnutrição: IMC para mães (variável categórica: IMC ≥25 kg/m^2^), baixa estatura para filhos (variável categórica: altura-para-idade: ≤2 escores-Z) (medidas aferidas).	IA grave foi associada a presença da Dupla Carga de Desnutrição. OR (IC95%) ajustada: 3,33 (1,41–7,84).	Forte
Vicenzi et al.^32^	Crianças >5 anos. São Leopoldo (RS).	Dados primários e amostra não probabilística.	Regressão de Poisson, com ajuste robusto para variância. Associação entre IA (sim ou não) e menor probabilidade de excesso em escolares. Excesso de peso: IMC-para-estatura (variável categórica: IMC-para-estatura: >1 escore-Z). (medida aferida).	IA foi associada a menor probabilidade de excesso em escolares. PR (IC95%) ajustada: 0,78 (0,64–0,96).	Moderada
Poblacion et al.^33^	Crianças <5 anos. Brasil (áreas urbanas e rurais).	Dados secundários e amostra probabilística (PNDS).	Regressão de Poisson, com ajuste robusto para variância. Associação entre IA (SA x IA moderada/grave) e baixo peso em crianças <5 anos. Baixo peso: peso-para-idade (variável categórica: peso-para-idade: ≤2 escore-Z) (medida aferida).	Baixo peso foi associado a IA moderada/grave em crianças <5 anos. PR (IC95%) ajustada: 1,4 (1,1–1,7) p=0,008.	Forte
Vasconcelos et al.^9^	Adultos e idosos com diagnóstico de HAS e/ou DM atendidos em UBS. Maceió (AL).	Dados primários e amostra probabilística.	Regressão logística simples. Associação entre IA (sim ou não) e menor prevalência de obesidade central e hipertrigliceridemia em usuários de UBS. Obesidade central: CC (variável categórica: sim ou não) (medida aferida). Hipertrigliceridemia: Triglicerídeos >200 mg/dL (variável categórica: sim ou não) (medida aferida).	Menor prevalência de adiposidade central e hipertrigliceridemia em usuários de UBS foi associada a IA. Hipertrigliceridemia: OR (IC95%): 0,74 (0,67–0,82), p=0,001. Adiposidade central: OR (IC95%): 0,12 (0,29–0,52), p=0,004.	Forte

Todos os artigos incluídos foram do tipo transversal. QATFQS: *Quality Assessment Tool For Quantitative Studies*; IA: insegurança alimentar domiciliar mensurada pela Escala Brasileira de Insegurança Alimentar; SA: segurança alimentar domiciliar mensurada pela Escala Brasileira de Insegurança Alimentar; IMC: índice de massa corpórea; PR: *Prevalence ratio*; IC: intervalo de confiança; p: valor de p (significância estatística p<0.05); PNDS: Pesquisa Nacional de Demografia e Saúde; POF: Pesquisa de Orçamentos Familiares; IBGE: Instituto Brasileiro de Geografia e Estatística; OR: *odds ratio*; DM: diabetes mellitus; HAS: hipertensão arterial sistêmica; UBS: Unidade Básica de Saúde; CC: circunferência da cintura.

Para fins de análise, os artigos incluídos categorizaram a EBIA em: 2 categorias (SA x IA, 9 artigos; SA/IA leve x IA moderada/grave, 4 artigos; SA x IA moderada/grave, 2 artigos; SA x IA moderada, 1 artigo; SA x IA leve, 1 artigo; SA/IA leve/moderada x IA grave, 1 artigo), 3 categorias (SA x IA leve x IA moderada/grave, 3 artigos) e 4 categorias (SA x IA leve x IA moderada x IA grave, 6 artigos).

Em relação à associação da IA com as DCNTs, a Tabela 1 apresenta a síntese das características gerais e dos principais resultados dos artigos incluídos que apresentaram associações. Os estudos revelaram a associação da IA com sobrepeso e excesso de peso/obesidade em diferentes faixas de idade do ciclo da vida e entre homens e mulheres, avaliada principalmente por meio do IMC. No tocante a como a variável de IA foi utilizada nas análises, 18 estudos a utilizaram como variável de exposição/fator de risco^5,22–24,26–28,31,32,34–42^ e 9 estudos como variável desfecho^9,25,29,30,33,43–46^.

Em adolescentes, os estudos encontraram associação de excesso de peso e IA grave^5^ e associação entre IA grave^22^, IA moderada/grave^23^ e SA/IA leve^24^ com excesso de peso. Encontrou-se também associação do sobrepeso com a IA (SA x IA) em mulheres^25^ e em adultos e idosos, independente do sexo^26^.

Sobre a obesidade, encontrou-se associação desta condição com todos os níveis de IA^27^ e IA moderada^5^ em mulheres. Ademais, observou-se associação da variável de IA moderada^28^ com obesidade em mulheres. Também foi identificada associação da obesidade com a IA entre os responsáveis pelo domicílio^29^ e associada à IA leve em idosos^30^. Além disso, foi percebida associação da IA grave com a dupla carga da má nutrição, ou seja, mãe com sobrepeso e filhos com baixa estatura no mesmo domicílio^31^.

Por outro lado, a menor prevalência de obesidade abdominal e hipertrigliceridemia em usuários com HAS e/ou DM atendidos em UBS foi associada a IA^9^ e a menor prevalência de sobrepeso e obesidade foi associada a IA moderada e grave em homens^29^. Já a IA foi associada a menor probabilidade de ter excesso de peso em escolares maiores de 5 anos^32^, e o baixo peso foi associado a IA moderada/grave em crianças menores 5 anos^33^.

Dos artigos incluídos, 13 não demonstraram associação da IA com os indicadores relacionados às DCNTs^34–46^, sendo 8 deles realizados com crianças e adolescentes^37–43,46^. Esses artigos estão dispostos em material suplementar.

Com as hipóteses e justificativas utilizadas pelos autores dos estudos incluídos para explicar a associação da IA com a obesidade, foi proposto um modelo teórico com os principais caminhos dessa associação, apresentados na Figura 2.

**Figura 2:** Modelo conceitual dos caminhos utilizados nos artigos incluídos para explicar a associação da insegurança alimentar com a obesidade e as demais doenças crônicas não transmissíveis. Revisão sistemática, 2023.

## DISCUSSÃO

O objetivo principal desta revisão sistemática foi utilizar-se do bem-sucedido histórico de estudos sobre a IA para determinar e documentar evidências sobre a associação da IA com as DCNTs no contexto da população brasileira. Os achados demonstram a existência de uma associação da IA com o sobrepeso em adolescentes, adultos e idosos de ambos os sexos e com a obesidade em mulheres e idosos.

A associação da IA com as DCNTs tem sido abordada em revisões sistemáticas. Miguel et al.^47^ estudaram a relação da IA com os fatores de riscos cardiometabólicos em adultos e idosos. A revisão incluiu 11 estudos com medidas *proxy* para DCNTs, mostrando a existência de uma associação direta da IA com condições como excesso de peso, HAS, DM, dislipidemias e estresse. No tocante à DM, Abdurahman et al.^48^ identificaram que a IA esteve associada à DM do tipo 2, porém, destacaram que no Canadá a escolha do instrumento de medição IA influencia o efeito sobre a chance de desenvolver a DM. Já Beltrán et al.^49^ não encontraram associação da DM com a IA. Para as doenças cardiovasculares, Liu e Eicher-Miller^50^ observaram associação da IA com HAS, doença cardíaca coronariana e ataque cardíaco em norte-americanos maiores de 17 anos.

Beltrán et al.^51^ encontraram associação da IA em adultos com a HAS autorreferida, mas essa associação não foi encontrada quando os autores realizaram o diagnóstico por meio da medida da pressão arterial ou revisão de prontuários. Arenas et al.^52^ estudaram a associação da IA com dislipidemias, e os resultados não revelaram associação da IA com dislipidemia aferida por dosagem lipídica laboratorial, porém os indivíduos em IA foram mais propensos a autorrelatar diagnósticos prévios de dislipidemia. Por fim, no cenário internacional, a associação da IA com a obesidade também foi documentada em revisões sistemáticas prévias^53–56^.

Nesta revisão, dedicada a estudar a população brasileira, apesar do amplo espectro de busca, a maioria expressiva dos estudos disponíveis abordou o estado nutricional e as análises da condição de sobrepeso e obesidade relacionada com a IA, destacando, assim, o pouco volume de estudos com população em risco ou acometida por HAS, DM, dislipidemias e doenças cardiovasculares. Também se observou a pouca diversidade de indicadores de risco, tendo a maioria dos estudos utilizado apenas o IMC.

Assim como observado nesta revisão, outras revisões documentaram a associação da IA com o excesso de peso/obesidade na população brasileira. Morais et al.^57,58^ observaram associação da IA com o excesso de peso e a obesidade na população brasileira, porém, no que se refere à população rural, Trivellato et al.^59^ não encontraram associação entre estado nutricional (excesso de peso, baixo peso e/ou desnutrição crônica) e IA.

A Figura 2 ilustra um modelo teórico contendo os principais caminhos utilizados pelos autores dos estudos incluídos para explicar a associação da IA com a obesidade, sendo eles:

1. A má nutrição;
2. Os mecanismos de enfrentamento e de convivência com a IA; e
3. O sedentarismo.

O caminho mais utilizado pelos autores para explicar essa associação foi o da má nutrição, ou seja, em famílias que vivenciam a IA há a aquisição e o consumo de alimentos de baixo custo, como os alimentos processados e ultraprocessados, e, consequentemente, o aumento da ingestão energética, de açúcares, gorduras e sal.

O segundo caminho mais utilizado pelos autores está embasado na existência de mecanismos de enfrentamento e de convivência com a IA dentro das famílias, como o ciclo de fome-fartura, caracterizado por períodos restritivos de subconsumo seguidos de períodos compensatórios de superconsumo. Tal mecanismo predispõe alterações metabólicas e psicológicas como medo, incerteza, restrição, privação, ansiedade e depressão, configurando quadros de estresse agudos e crônicos que alteram o comportamento alimentar e favorecem o desenvolvimento da obesidade.

O caminho menos utilizado foi o do sedentarismo, que teoriza que o estado de inatividade física de pessoas em IA, decorrente da situação socioeconômica, favorece o desenvolvimento da obesidade. Nos três caminhos utilizados nos artigos incluídos, a obesidade figura como fator que media o processo de desenvolvimento das demais DCNTs.

Em concordância, em nível internacional, os pesquisadores também utilizam esses caminhos para explicar a associação^53,54,60,61^. A relação da IA com a má nutrição está documentada na literatura. Autores descrevem que a presença da IA está acompanhada da menor variedade de alimentos, da maior ingestão energética e de dietas monótonas, ou seja, não se trata apenas da ausência de alimentos, mas também do acesso a alimentos de baixa qualidade^47,57,62,63^.

Estudos apontam a presença da obesidade como o principal caminho e mediador que conecta a IA ao desenvolvimento das demais DCNTs, como DM e HAS. Porém, autores destacam a importância de se analisar os potenciais efeitos negativos da IA para a saúde, independente da obesidade^50,64,65^.

Cabe destacar a necessidade de adensar o debate sobre a associação da IA com as DCNTs, incorporando aos estudos variáveis relacionadas às desigualdades sociais, e no que se refere ao acesso à saúde, às condições de trabalho e ao ambiente alimentar, temas que contribuem para ampliar o contexto de discussão e a rede de fatores que importam para o debate, tanto da IA como das DCNTs — especialmente no cenário brasileiro, marcado pela profunda desigualdade social e regional.

Desse modo, a associação entre IA e DCNTs é complexa. O surgimento de uma condição crônica, como a obesidade, a DM e a HAS, é resultado de um conjunto amplo de fatores determinantes e também está relacionado a uma exposição prévia e contínua a esses determinantes. No entanto, destaca-se que nos estudos incluídos na revisão, o tempo de exposição da população à IA não foi considerado, devido à natureza transversal dos estudos.

Assim, a ausência de estudos longitudinais que analisem o desenvolvimento de DCNTs a partir da exposição à IA é uma lacuna científica importante. Por outro lado, os estudos estabeleceram a associação entre IA e o risco/presença de DCNTs, ou seja, indivíduos que convivem com essas condições também sofrem com a privação alimentar, contexto que se expressa como um importante limitante da implementação e adoção de medidas de cuidado e tratamento dessas condições crônicas.

Vale ressaltar que, em nível global, os métodos de mensuração de IA diferem entre os países/regiões, especialmente no tempo de referência da IA, dificultando também a comparação das evidências. Outra complexidade relacionada à análise da IA e DCNTs está no contexto social e econômico de cada país, de forma que diferenças nos sistemas agroalimentares, níveis de desenvolvimento e o estado da transição nutricional devem ser considerados nos estudos futuros e para a comparação com os resultados observados no Brasil.

Os artigos incluídos nesta revisão não produziram evidências sobre outras DCNTs de interesse para a saúde pública no Brasil, como a DM e a HAS, evidenciando uma importante lacuna científica e a necessidade da construção de uma agenda de pesquisa no tema que priorize estudos longitudinais, na perspectiva de gerar evidências de causalidade na relação da IA com as DCNTs.

As evidências aqui geradas reforçam que o acesso aos alimentos deve ser garantido, com práticas promotoras de saúde, e fornecem subsídios para a focalização de intervenção e programas em saúde pública com vistas a combater a IA e promover, prevenir e controlar as DCNTs. Os serviços de saúde devem estar atentos à alta prevalência da IA na população brasileira e a como esse cenário pode implicar no cuidado ofertado aos indivíduos acometidos por DCNTs. Os investimentos nas intervenções e os programas públicos relacionados ao tema da associação da IA com as DCNTs vão de encontro aos Objetivos do Desenvolvimento Sustentável da ONU, assumidos pelo Brasil para a Agenda 2030.

Portanto, apesar do seu bem-sucedido histórico de estudos sobre IA, no Brasil a associação da IA com as DCNTs permanece pouco abordada. Os estudos revisados corroboram evidências de associação da IA com o sobrepeso em todas as faixas etárias, a obesidade entre mulheres e idosos. Assim, são necessárias intervenções públicas e uma agenda forte de combate à fome aliada com práticas promotoras de saúde e cuidado nutricional, especialmente na Atenção Primária à Saúde. Sugerem-se investimentos em estudos que abordem a temática da associação da IA com as DCNTs, além da obesidade, principalmente com desenho longitudinal.
